# High-Level Expression of Palmitoylated MPP1 Recombinant Protein in Mammalian Cells

**DOI:** 10.3390/membranes11090715

**Published:** 2021-09-17

**Authors:** Agnieszka Chytła, Weronika Gajdzik-Nowak, Agnieszka Biernatowska, Aleksander F. Sikorski, Aleksander Czogalla

**Affiliations:** 1 Department of Cytobiochemistry, Faculty of Biotechnology, University of Wroclaw, 50-383 Wroclaw, Poland; agnieszka.chytla@uwr.edu.pl (A.C.); weronika.gajdzik@uwr.edu.pl (W.G.-N.); 2 Research and Development Center, Regional Specialist Hospital, Kamieńskiego 73a, 51-154 Wroclaw, Poland; aleksander.sikorski@wssk.wroc.pl

**Keywords:** MPP1, palmitoylation, membrane rafts, HEK-293F, transfection, purification, Acyl-RAC

## Abstract

Our recent studies have pointed to an important role of the MAGUK family member, MPP1, as a crucial molecule interacting with flotillins and involved in the lateral organization of the erythroid plasma membrane. The palmitoylation of MPP1 seems to be an important element in this process; however, studies on the direct effect of palmitoylation on protein–protein or protein–membrane interactions in vitro are still challenging due to the difficulties in obtaining functional post-translationally modified recombinant proteins and the lack of comprehensive protocols for the purification of palmitoylated proteins. In this work, we present an optimized approach for the high-yield overexpression and purification of palmitoylated recombinant MPP1 protein in mammalian HEK-293F cells. The presented approach facilitates further studies on the molecular mechanism of lateral membrane organization and the functional impact of the palmitoylation of MPP1, which could also be carried out for other palmitoylated proteins.

## 1. Introduction

Membrane-associated guanylate kinases (MAGUKs) are widely expressed proteins in all types of eukaryotic cells [1]. This well-conserved group of scaffolding proteins includes several subfamilies that are involved in multiple cellular processes, such as cell polarity, cell signalling, and cell adhesion [2,3]. One of these subfamilies is named the membrane palmitoylated proteins (MPPs). MPPs are ubiquitously expressed, membrane-associated, palmitoylated proteins found at the cytosolic leaflet of the plasma membrane (PM) [4]. Similar to other MAGUKs, they contain a highly conserved core of postsynaptic density protein 95/disc large/zonula occludens-1 (PDZ) domain, an Src homology 3 (SH3) domain, and a catalytically inactive guanylate kinase (GUK) domain. Another characteristic domain shared within some members of the MPP family is the D5 domain, also known as HOOK [4,5]. One of the most thoroughly examined members of this group, MPP1, was first described in red blood cells (RBCs) and is primarily known for its role in maintaining the proper shape of erythrocytes by participating in the formation of a ternary complex together with the 4.1R protein and glycophorin C [6,7,8]. Importantly, our previous studies demonstrated the key role of MPP1 in the lateral organization of the erythroid cell membrane [9,10,11,12]. Experiments performed on the human erythroleukemia cell line (HEL), which are erythrocyte progenitor cells, showed that silencing MPP1 expression or the inhibition of its palmitoylation significantly reduced the order parameters of both PM or PM-derived vesicles in comparison to control cells [9,13]. Moreover, a reduction in MAP kinase signalling pathway activation via raft-dependent RTK receptors and their downstream signalling pathways was also observed [11]. Thus, these studies emphasized that MPP1 acts as a specific “organizer” of the lateral membrane and influences the formation of functional membrane rafts at the PM of erythroid cells. Although the relevance of membrane rafts, defined as heterogeneous, dynamic protein–lipid nanodomains in the spatio–temporal compartmentalization of membrane components, is well characterized [14,15] the physiological factors driving their organization are still poorly characterized. On the other hand, it has become evident that the lateral organization of biological membranes into specified domains depends on the cooperative interaction/clustering of protein and lipids (for a review, see [15]). Specific endogenous membrane-organizers have therefore been intensively researched. In this particular context, to understand the MPP1-driven membrane raft formation, our further studies of the MPP1 interactome have led to the identification of novel protein partners of MPP1, the raft protein markers flotillin 1 and flotillin 2 [10]. Flotillins represent the major structural scaffolding proteins in erythroid raft domains [16]. Due to their oligomeric nature, flotillins serve as assembly sites enabling the association of different molecules, thus actively contributing to numerous cellular processes (e.g., endocytosis, signalling) in the PM [17,18,19,20]. Most recently, using a surface plasmon resonance-based approach and recombinant proteins overexpressed in bacterial cells, we determined the kinetic parameters for the MPP1–flotillin 1/flotillin 2 interactions and showed that these proteins form high-affinity complexes in vitro. Moreover, using molecular dynamics simulations and a series of MPP1-truncated mutants, we identified a flotillin binding site within the MPP1 D5 domain [21]. Based on our findings and models presented by others [22], we hypothesize that MPP1 triggers the clustering of flotillins-based assemblies into functional membrane raft domains in the PM [10,21]. Such a concept also fits the model ascribing the dominant role of scaffolding proteins in capturing rafts-associated elements [23]. However, to obtain a full picture of the mechanism, one important issue related to MPP1 palmitoylation needs to be further addressed. Palmitoylation is an important lipid modification that relies on the attachment of palmitate to internal cysteine residues via a thioester linkage [24]. In the case of MAGUK proteins, palmitoylation is fundamental, enabling the recruitment and association of other molecules and the formation of multi-component complexes at the cytosolic leaflet of the PM. This has been well documented in neurons, where the palmitoylation of the PSD-95 (postsynaptic density 95) protein determines the clustering of AMPA (α-amino-3-hydroxy-5-methylisoxazole-4-propionic acid) receptors and, thus, provides proper synaptic trafficking and neuron activity [25,26,27]. On the other hand, the palmitoylation of PSD-95 was found to be significant for the stabilization of cholesterol and sphingomyelin-enriched domains during neuronal development [28].

MPP1 is the major palmitoylated protein in RBC membranes [6] and the inhibition of its palmitoylation was shown to correlate with changes in lateral PM organization in erythroid cells [9]. To study the molecular mechanisms of this issue, in vitro studies on the direct effect of palmitoylated proteins using model membranes are required but also challenging. While it is possible to obtain a high yield of recombinant MPP1 protein from *Escherichia coli* cells [5,10,21,29], the bacterial system cannot be used to overexpress the protein with crucial post-translational modifications (PTM), such as palmitoylation, because it lacks palmitoyl acyltransferases or related enzymes. On the other hand, overexpression in mammalian systems offers molecular chaperones and co-factors that allow for the production of correctly folded proteins with the desired PTMs, which are important for their functionality and full activity [30,31]. So far, most reports describe the purification of glycosylated proteins [32,33,34]; however, a detailed protocol describing the step-by-step overexpression and purification of palmitoylated recombinant proteins is currently missing.

In this study, we optimized the transient expression of the MPP1 protein in HEK FreeStyle™ 293-F cells (HEK-293F). To the best of our knowledge, this is the first work describing the purification of “in cellula” palmitoylated protein overexpressed in the mammalian system. The presented study not only facilitates further research on the functional impact of palmitoylated MPP1 on PM organization in vitro but may also serve as a primer for the overexpression and purification of other recombinant palmitoylated peripheral membrane proteins.

## 2. Materials and Methods

### 2.1. Plasmid/DNA

An expression vector carrying a sequence encoding fused protein, mEGFP-MPP1 (Figure S1), was prepared by cloning the MPP1 sequence (NCBI Reference Sequence: NM_002436.3) into the mEGFP-HRas plasmid (Addgene Plasmid #18662) using XhoI and NotI restriction sites (Table S1).

A pcDNA3.1 Hygro-ACP_His-tag_MPP1_TAP-tag expression vector was constructed and synthesized commercially (GenScript). The MPP1 sequence was flagged by an ACP-tag (Acyl Carrier Protein) and a His-tag at the N-terminus and a TAP-tag at the C-terminus. A Tobacco Etch Virus (TEV) and an enterokinase recognition site (EK) precede the MPP1 sequence, and a 3C protease recognition site precedes the TAP-tag. The ACP-tag sequence plus the sequence recognized by the TEV protease were deleted using site-directed mutagenesis (Q5^®^ Kit, NEB) (Table S1). The resulting construct containing a His-tag and TAP-tag only was used in further work (Figure S1a,b).

### 2.2. Cell Culture and Transfection

The FreeStyle™ 293-F cells (HEK-293F, Thermo Fisher Scientific, Waltham, MA, USA) were grown according to the manufacturer’s instructions in 125 mL or 500 mL Nalgene™ Erlenmeyer flasks with plain bottoms and vented caps (Thermo Fisher) in the FreeStyle™ medium at 37 °C in an 8% CO_2_ atmosphere with gentle shaking (130 rpm, Infors HT Minitron).

One day before the transfection, cells were sub-cultured to a density of 10^6^ cells/mL. On the day of transfection, cells were centrifuged at 200× *g* at RT for 5 min, resuspended in fresh medium (10 mL for optimization experiments in 25 cm^2^ Nunc™ EasYFlask™ or 500 mL for overexpression in 2 L flasks) at a density of 10^6^ cells/mL, and transfected using DNA and 25 kDa linear polyethyleneimine (PEI, Polysciences, Warrington, PA, USA) polyplexes. Briefly, the working solutions of the DNA and working solutions of the PEI were prepared separately in sterile 150 mM NaCl, then mixed and incubated at room temperature for 10 min. After the incubation, DNA–PEI polyplexes were added to the cell culture (0.5 µg DNA per 10^6^ cells). The final volume ratio of polyplexes and cell culture medium was 1/10. Depending on the experiment, cells were incubated with DNA–PEI either for 17 h at 31 °C, or 5 h at 37 °C, then the cells were centrifuged at 200× *g*, RT for 5 min and resuspended in the same volume of a fresh medium to remove any remaining polyplexes or free PEI, which is toxic to cells [35]. After centrifugation, cells transfected at 31 °C were returned to the incubator and cultured for an additional 48 h. As for the cells transfected for 5 h at 37 °C, they were cultured for another 17 h at 37 °C for regeneration, followed by incubation at 31 °C for another 48 h. The temperature change from 37 °C to 31 °C was intended to induce mild hypothermia, since it is supposed to stimulate protein overexpression in mammalian systems [36].

### 2.3. Transfection Analysis

For the optimization of the transfection yield, cells were transfected with the plasmid encoding mEGFP-MPP1, as described above. Samples containing 3 × 10^5^ cells were harvested after selected time points (24 h, 48 h, and 72 h post-transfection), centrifuged (5 min, 200× *g*), and resuspended in 500 µL phosphate buffer (PBS; Gibco, Thermo Fisher Scientific, Amarillo, TX, USA). Data acquisition and analysis was performed using a NovoCyte Flow Cytometer (excitation laser: 488 nm, detection channel equipped with bandpass emission filter 530/30 nm) by collecting 10,000 events for each sample. A TC10™ Automated Cell Counter (Bio-Rad, Hercules, CA, USA) was used to determine the total cell count and cell viability via the Trypan blue dye exclusion method.

### 2.4. Purification of MPP1

MPP1-transfected cells obtained from 2 L (4 × 500 mL) culture were harvested after 72 h post-transfection, centrifuged at 2000× *g*, 4 °C for 5 min, and resuspended in cold 50 mM HEPES, pH 7.4, 400 mM NaCl (RES buffer), supplemented with protease inhibitors (cOmplete™ Protease Inhibitor Cocktail (Roche, Basel, Switzerland): namely, 1 tablet per 100 mL of buffer; 5 µM E64 (Sigma-Aldrich, St. Louis, MO, USA); 100 µg/mL Pefabloc^®^ SC (Roche)). Then, the cell suspension was passed through a Microfluidizer LM20 (Microfluidics International Corporation) at 5000 PSI for three cycles. Next, the collected suspension (cell lysate) was subjected to centrifugation at 500× *g* for 10 min at 4 °C to remove the nuclear fraction and cell debris (P1). After centrifugation, the supernatant (S1) fraction was incubated with 2% CHAPS for 2 h at 4 °C. After the incubation solubilized, S1 was incubated with IgG 6 Sepharose FastFlow (GE Healthcare, Chicago, IL, USA) and pre-equilibrated with a RES buffer for 2 h at 4 °C. After the incubation, the resin was loaded on the column and washed with wash buffers (WB) in sequence: WB (1× PBS pH 7.4; 300 mM NaCl, 1% CHAPS, 5% glycerol), WB + 5 mM ATP 10 mM MgCl_2_, WB, and WB + 2 mM EDTA. The final step was performed using an elution buffer (EB; 1× PBS pH 7.4, 150 mM NaCl, 0.5% CHAPS, 10% glycerol). The recombinant MPP1 protein was cleaved off from the resin using 0.04 mg/mL GST-tagged 3C protease (MPI-CBG protein facility, Dresden, Germany) through overnight incubation at 4 °C. Nine 1 mL fractions were collected and incubated with Glutathione-Sepharose 4B beads (GE Healthcare) for 1 h at 4 °C to remove the 3C protease from the MPP1 protein solution. The final clean-up of the MPP1 was carried out using PureCube 100 INDIGO Ni-Agarose (Cube Biotech, Monheim am Rhein, Germany). Fractions with the highest absorbance at λ = 280 nm were collected and either directly dialyzed for further experiments or snap-frozen in liquid nitrogen and stored at −80 °C.

### 2.5. Detection of S-Palmitoylation

S-palmitoylation of the recombinant MPP1 protein was identified by the acyl-resin assisted capture (Acyl-RAC) method using a CAPTUREome™ S-Palmitoylated Protein Kit (Badrilla, K010-310; Leeds, UK). For the experiment, 2 mg of total protein from MPP1-transfected cell lysates and 100 µg of purified MPP1 recombinant protein were incubated in a blocking buffer and then incubated at 40 °C for 4 h. Further steps of the procedure were carried out according to the manufacturer’s protocol. Finally, the obtained samples, which contained palmitoylated proteins only, were subjected to SDS–PAGE separation and Western blot analysis using specific antibodies.

### 2.6. SDS–PAGE and Western Blot

The purity of the recombinant MPP1 protein was checked using SDS–PAGE and Western blot analysis or Coomassie staining. Proteins were separated on 10% Tris-Glycine SDS–PAGE gel and electrotransferred on a nitrocellulose membrane (Amersham™ Protran^®^) or fixed in 50% methanol. The membrane was blocked with 5% skim milk in TBS-T (20 mM Tris, pH 7.4; 150 mM NaCl, 0.05% Tween-20) overnight at 4 °C, then incubated with primary antibodies (Anti-His-tag Antibody (AD1.1.10) or Anti-GAPDH Antibody (6C5), Santa Cruz Biotechnology, Dallas, TX, USA) in TBS-T overnight at 4 °C with secondary antibodies conjugated with HRP (Peroxidase AffiniPure Goat Anti-Mouse IgG (H + L), Jackson Immuno Research) in TBS-T for 1 h at room temperature. Between each incubation step, membranes were washed with TBS-T. The blots were developed with Radiance ECL (Azure Biosystems, Dublin, CA, USA) and imaged with the Azure image system (Azure 600; Azure Biosystems). The methanol-fixed gels were stained with Coomassie Brilliant Blue G-250, then washed with distilled water and imaged with the Azure image system.

### 2.7. Circular Dichroism (CD) Spectroscopy

Purified recombinant MPP1 was dialyzed against an SLB buffer (10 mM HEPES pH 7.4, 150 mM NaCl). Measurements were carried out with a J-1500 (JASCO) spectropolarimeter using a 1 mm path length cell in a temperature range between 10 °C and 70 °C, at three time points (1 day, 3 days and 7 days after dialysis). The spectra were collected at wavelengths ranging from 205 to 260 nm. The mean residue ellipticity was calculated from the equation:

(1) $$\emptyset =\frac{\mathrm{CD}\;\mathrm{unit}}{10*{\mathrm{residues}*\mathrm{pathlength}*C}_{m}},$$

where CD units are in mdeg and C_m_ is molar concentration. Thermal denaturation was calculated from the equation:

(2) $$Fu=\frac{\emptyset_{T}-\emptyset_{\min\left(10{}^{\circ}C\right)}}{\emptyset_{\max\left(70{}^{\circ}C\right)}-\emptyset_{\min\left(10{}^{\circ}C\right)}},$$

where ∅_(min)_ is the mean residue ellipticity at 222 nm at minimum temperature, ∅_(max)_ is the mean residue ellipticity at 222 nm at maximum temperature, and ∅_T_ is the mean residue ellipticity at 222 nm for the tested temperature_._

## 3. Results and Discussion

### 3.1. Optimization of HEK-293F Cells Transfection and MPP1 Overexpression

One of the key steps in the production of recombinant proteins in mammalian cells is the optimization of cell transfection conditions in order to obtain the best possible efficiency of overexpression while maintaining a reasonable cell viability. These parameters should be established empirically for each cell line, as no universal conditions are recognized so far. In most cases, transfection efficiency depends mainly on the DNA:PEI ratio [35,37,38,39,40]. PEI is a cationic polymer that is commonly used in transient gene expression (TGE) as a transfection reagent due to its known advantages (low costs, high transfection yields, and being easy to scale up with transfection) [38,41]. There are several hypotheses for how PEI introduces DNA into a cell’s interior; however, all of them share the assumption that positively charged PEI interacts with negatively charged DNA to form a net-positively charged complex. These complexes can then bind to negatively charged cellular surfaces, then be transported to the cytoplasm and subsequently to the nucleus. Despite the differences in hypotheses regarding the key steps in the molecular mechanisms underlying the successful transfection of cells via DNA:PEI complexes, all agree that the translocation of the DNA from the cytoplasm to the nucleus is crucial for the transfection process [35,42,43,44].

Apart from the DNA:PEI ratio, the medium used for the complexation is equally important, as some components present in cell media may interfere with the delivery of the DNA:PEI polyplexes or change their properties [45,46]. Instead of testing different transfection media, this obstacle can be easily overcome with the use of 150 mM of NaCl. The role in the formation and stabilization of the DNA:PEI polyplexes is mostly due to the effect Na^+^ and Cl^−^ ions have on electrostatic interactions that stabilize polyplexes [47,48]. The latter formed in NaCl were found to be bigger and more stable than those formed in other conditions, which positively affected the transfection efficiency [47,48,49,50].

**Figure 1:**
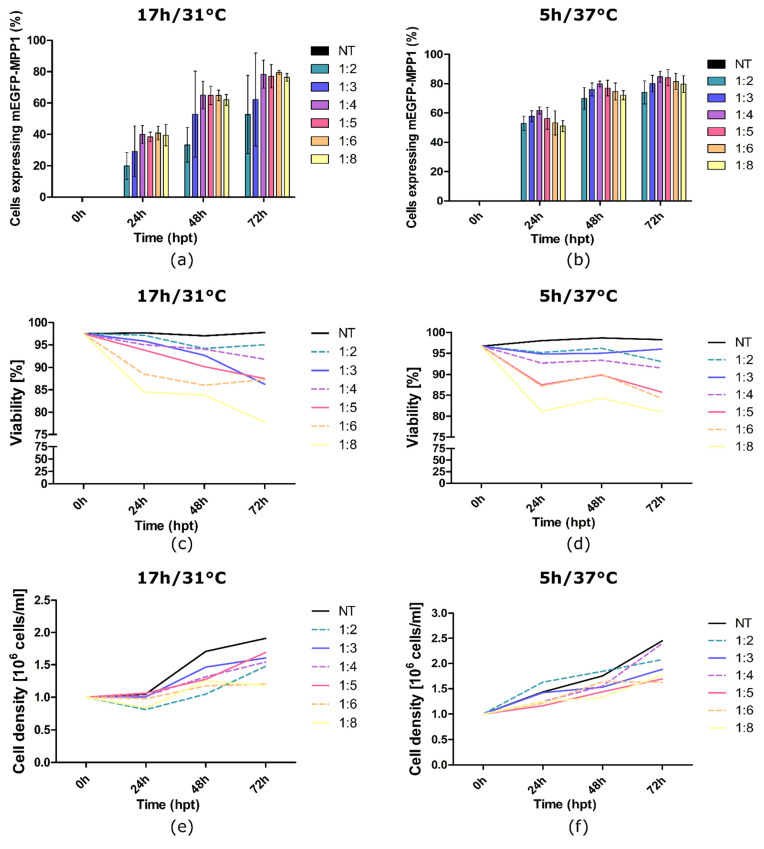
The effect of time, temperature, and DNA/PEI ratio on mEGFP-MPP1 overexpression and HEK-293F cell viability and density. Flow cytometry analysis of the level of mEGFP-MPP1 overexpression after (**a**) 17 h transfection at 31 °C or (**b**) 5 h transfection at 37 °C using different DNA:PEI ratios. Error bars represent SD. Trypan blue-based analysis of the viability of cells transfected for (**c**) 17 h at 31 °C or (**d**) 5 h at 37 °C with different DNA:PEI ratios. The density of cells transfected for (**e**) 17 h at 31 °C or (**f**) 5 h at 37 °C with different DNA:PEI ratios. NT—non-transfected cells/ control; hpt—hours post-transfection. The procedure was performed in triplicate.

Another factor that can affect the transfection and overexpression process is the temperature during the cell culture. The mild hypothermic conditions are beneficial for recombinant protein overexpression due to cell cycle arrest and the switching of the cell towards protein synthesis; however, they may not be optimal for the transfection step [36]. Thus, in the optimization process, we focused on three crucial parameters: (1) time, (2) temperature of the transfection and (3) the DNA:PEI weight ratio. To monitor the influence of the above mentioned parameters on the expression of the mEGFP-MPP1 protein in HEK-293F cells, a fluorescent mEGFP-MPP1 construct was used, which enabled the analysis using flow cytometry. For this purpose, cells were transfected using six different DNA:PEI weight ratios (1:2; 1:3; 1:4; 1:5; 1:6; 1:8), and the transfection was carried out for 5 h at 37 °C or 17 h at the reduced temperature of 31 °C. Post transfection, cells were either recovered for 17 h at 37 °C and then cultured at a reduced temperature of 31 °C (5 h/37 °C), or they were kept at 31 °C (17 h/31 °C). The level of mEGFP-MPP1 overexpression, cell density and viability were monitored after 24, 48 and 72 h (Figure 1).

Twenty-four hours post-transfection (24 hpt), we observed a nearly two-fold increase in mEGFP-MPP1 overexpression in cells transfected for 5 h at 37 °C compared to those transfected for 17 h at 31 °C (Figure 1a,b). On the last day of the overexpression (72 hpt), the total amount of overexpressed mEGFP-MPP1 was comparable in both cases (Figure 1a,b). The highest transfection levels were obtained for cells transfected for 5 h with 1:4 and 1:5 DNA:PEI ratios (84.73% and 84.14%, respectively). We observed that the cell viability decreased with a decrease in the DNA:PEI ratio. Still, cells treated with 1:2–1:5 DNA:PEI presented relatively high viability (Figure 1c,d). Cells subjected to mild hypothermic conditions from the beginning showed reduced proliferation. However, the combination of lower temperature with a prolonged exposition to DNA-PEI complex did not improve the overall expression efficiency. For the cells transfected at 37 °C and then transferred to the 31 °C 24 hpt, the slowest growth combined with acceptable viability is seen for the ratio 1:5 (Figure 1e). Taking into account the results discussed above and the fact that the target plasmid was slightly larger (Figure S1), we decided that a 5 h transfection at 37 °C and a 1:5 DNA:PEI ratio was the most suitable for our further experiments.

### 3.2. Protein Purification

**Figure 2:**
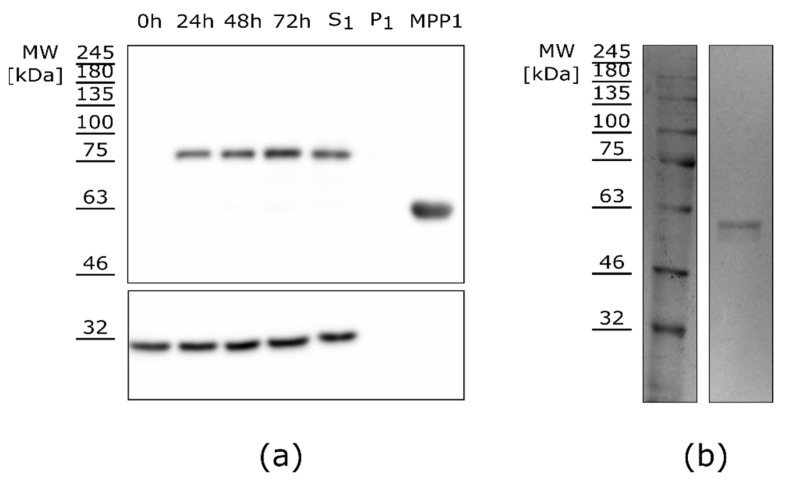
Expression and purification of MPP1 protein from HEK-293F cells. Western blot analysis of (**a**) crude cell lysates collected through transfection (0 h–72 h), samples collected after centrifugation (S1, P1) and purified recombinant protein (2 µg). The upper membrane was incubated with Anti-His-tag antibodies to detect overexpressed MPP1 and the lower membrane with Anti-GAPDH antibodies; (**b**) Coomassie blue staining of the SDS–PAGE gel with purified recombinant MPP1. The lower mass of purified recombinant MPP1 is a result of TAP-tag cleavage during the purification (for details, see Figure S1b).

The MPP1 protein was purified in native conditions using affinity chromatography towards a TAP-tag as described in the materials and methods. The level of the overexpressed protein was monitored through the increasing signal from the samples collected during the overexpression in HEK-293F cells (Figure 2a, 0–72 h). As MPP1 is a peripheral protein, the original idea was to fractionate the cell lysate into “cytosolic” and “membrane” fractions; however, as the protein level of MPP1 in these fractions was comparable (Figure S2), we decided to omit the fractionation step and instead purify the recombinant protein from whole cell lysate—see Figure S2. Both approaches exhibited a comparable purification efficiency. Overall, the optimized conditions of transfection and expression allowed the high-yield purification of recombinant MPP1 from HEK-293F cells. From a 2 L cell culture, we were able to obtain a total of 5 mg of recombinant protein.

### 3.3. Secondary Structure

**Figure 3:**
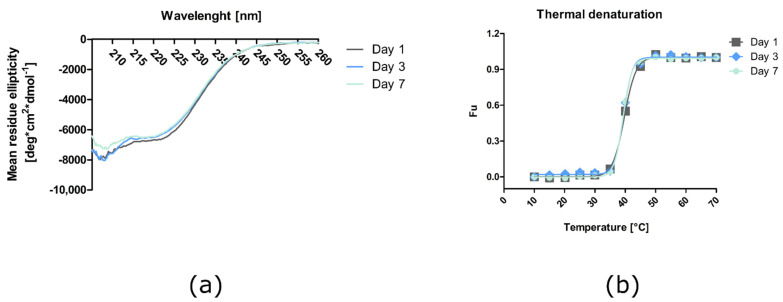
Characterization of the purified recombinant MPP1 protein using circular dichroism spectroscopy. (**a**) CD spectra of MPP1 protein 1, 3, and 7 days after dialysis recorded at 20 °C. (**b**) Thermal denaturation of MPP1 protein reflected via changes in ellipticity at 222 nm. The MPP1 was stored at 4 °C. Fu—fraction unfolded.

In the next step, the secondary structure of the purified MPP1 protein was analyzed using CD, including the analysis of protein thermal stability at selected storage-time points. The obtained CD spectra show a clear minimum at 208 nm and another around 220 nm, which suggests a mix of the alpha–helical and beta structure of the protein (Figure 3a). These results are compatible with our theoretical model [51], AlphaFold predictions [52], CD spectra-based structure analysis with the BeStSel algorithm [53], and the CD results obtained for the protein purified in the bacterial system [21]. The values calculated from the model indicate that these two secondary-structure types account for approximately 40% of the protein structure. Additionally, the comparative analysis of the collected spectra indicates that the protein stored at 4 °C remains stable even one week after thawing (Figure 3). The data from thermal melting curve analysis of the MPP1 protein for each sample were fitted to the Boltzmann sigmoid curve (GraphPad Prism, GraphPad Software Inc., San Diego, CA, USA) and the melting temperature was calculated to be approximately 39 °C (Figure 3b).

### 3.4. Analysis of S-Palmitoylation

The main goal of this study was to purify naturally palmitoylated recombinant MPP1 overexpressed in HEK-293F cells, which largely reflects the endogenous protein found in the PM of eukaryotic cells. The presence of this modification in the overexpressed lysates and purified MPP protein fraction was checked by the Acyl-RAC method [54]. Briefly, the method consists of three main steps: a blocking step, cleavage step, and capture step. The procedure started with the blocking of free thiol groups. After that, the remaining palmitate groups were removed with a thioester cleavage reagent (cleaved fraction, cF) or left untreated with a preservative (preserved fraction, pF). The proteins, with newly released thiol groups, were incubated with the capture resin. In the final step, the captured proteins (which represent the palmitoylated species) were eluted and then analyzed via a Western blot.

**Figure 4:**
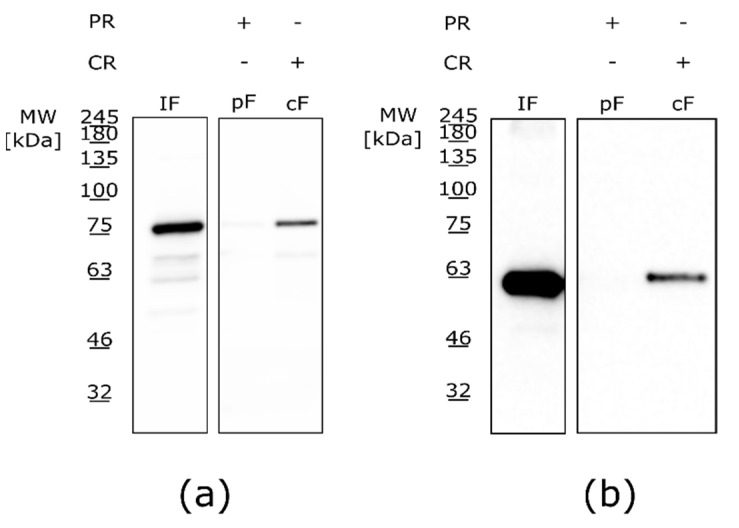
Identification of palmitoylation of overexpressed recombinant MPP1 protein by the Acyl-RAC method. Identification of palmitoylation of (**a**) overexpressed MPP1 protein in HEK-293F lysates and (**b**) recombinant MPP1 purified from HEK-293F cells. Both membranes were incubated with Anti-His-tag antibodies to detect recombinant MPP1. IF—input fraction (sample taken before the cleavage step); pF—preserved fraction; cF—cleaved fraction; PR—preservative reagent; CR—cleavage reagent.

The results obtained with the Acyl-RAC method confirmed that by using our optimized protocol we were able to overexpress and purify naturally palmitoylated recombinant MPP1 from HEK-293F cells (Figure 4). As can be observed, the entire pool of overexpressed MPP1 does not undergo palmitoylation; however, this may be due to the dynamic palmitoylation/depalmitoylation cycles that were previously shown for several endogenous palmitoylated proteins in the HEK-293T cells [55]. Such an issue might be further overcome by the use of APTs inhibitors—e.g., Palmostatin B—during overexpression and/or purification [56,57]. Another possibility to increase the palmitoylation level of recombinant proteins is the co-expression of recombinant protein with specific PAT/PATs enzyme(s) [57,58,59]. However, in the case of MPP1, such a task would be difficult to accomplish, as currently it is unknown which DHHC enzyme palmitoylates the MPP1 protein or whether any specificity of DHHCs towards MPP1 exists. In mammals, the family of PATs consists of 23 members and often more than one enzyme modifies a given protein; therefore, finding appropriate DHHC is rather challenging [59,60,61].

## 4. Conclusions

This study aimed to find the optimal transfection parameters for the maximized overexpression yield of naturally palmitoylated recombinant MPP1 in HEK-293F cells. Using our optimized protocol, we were able to obtain a good purification efficiency (5 mg/per 2 L of cell culture) of the recombinant MPP1 protein which, as shown by the Acyl-RAC method, is palmitoylated, well folded, and stable in solution. The presented approach may be considered as a showcase paving the way to further optimize the overexpression levels, purification yields, and palmitoylation levels of various peripheral membrane proteins. We believe that the protocol described above may be of great value for other researchers who intend to investigate the direct impact of palmitoylated proteins in vitro—e.g., in combination with membrane model systems in bottom-up reconstitution approaches.

## Supplementary Materials

The following are available online at https://www.mdpi.com/article/10.3390/membranes11090715/s1: Figure S1: Plasmid maps and recombinant protein schemes; Table S1: Primers used for cloning and mutagenesis; Figure S2: Expression and fractionation of MPP1 protein from HEK-293F cells.
