# Supplementary material for: High-Level Expression of Palmitoylated MPP1 Recombinant Protein in Mammalian Cells
Source: Membranes (Basel). 2021 Sep 17;11(9):715. doi: 10.3390/membranes11090715 (PMC8470630; doi:10.3390/membranes11090715)
Supplement: Supplementary file 1 [file membranes-11-00715-s001.zip › membranes-1368869-supplementary.pdf]

SUPPLEMENTARY INFORMATION:

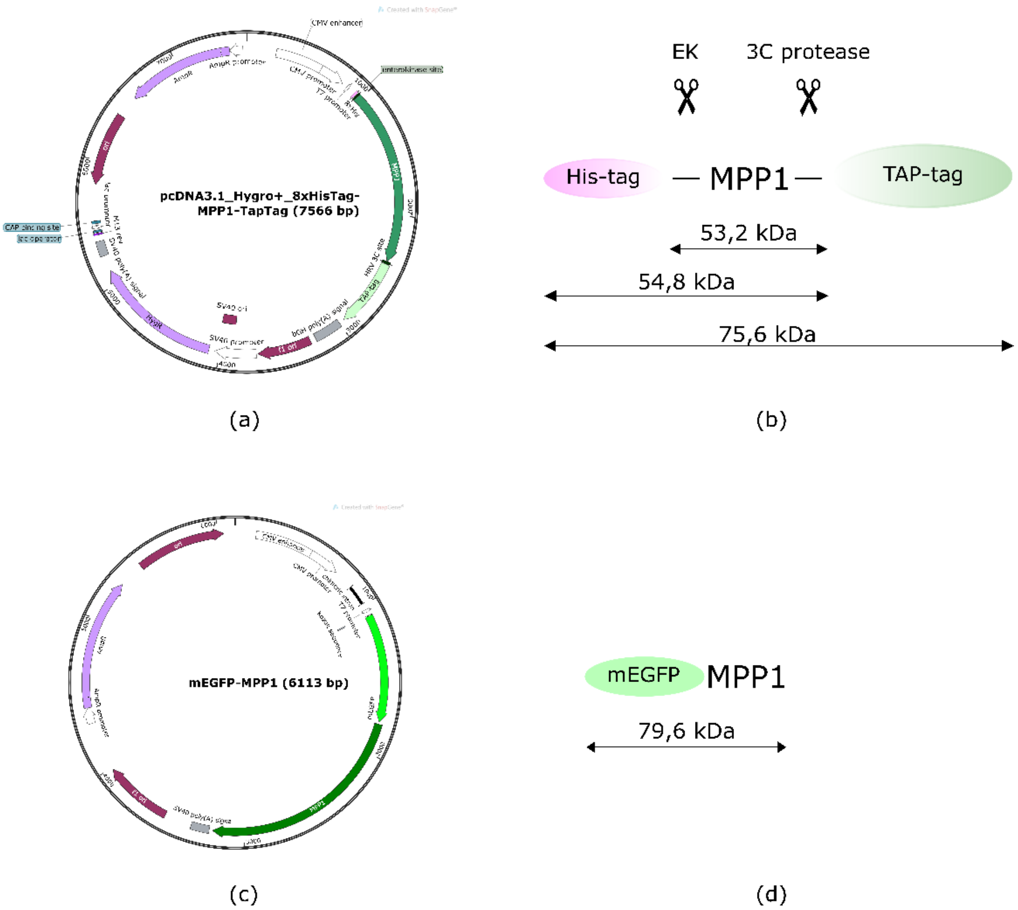

Figure S1. Plasmid maps and recombinant protein schemes (a) Map of a pcDNA3.1_Hygro+_8xHisTag-MPP1-Tap-tag plasmid vector used to overexpress recombinant MPP1 protein shown schematically on (b) with indicated proteases recognition sites. (c) Map of an mEGFP-MPP1 plasmid vector used to overexpress recombinant mEGFP-MPP1 protein shown schematically on (d).

Table S1. Primers used for cloning and mutagenesis

Primer	Sequence (5′ --> 3′)	
MPP1-XhoI-Fwd	TCGCTCGAGATGACCCTCAAGG	Cloning
MPP1-NotI-Rev	ATTGCGGCCGCTTAGTAAACCCA	Cloning
MPP1ΔACP-Fwd	CATCATCACCACCACCAC	Mutagenesis
MPP1ΔACP-Rev	CATGGATCCGAGCTCGGT	Mutagenesis

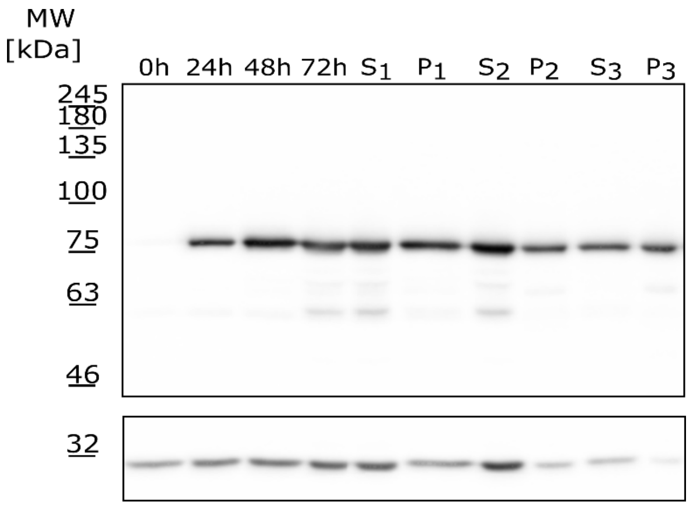

Figure S2. Expression and fractionation of MPP1 protein from HEK-293F cells. Western Blot analysis of the crude cell lysates collected through transfection (0h-72h) and samples at each step of fractionation (S1-P3; S – supernatant, P – pellet, 1-3 – number of centrifugations: 1) 1000 x g, 10 min; 2) 250 000 x g, 50 min; 3) 250 000 x g, 50 min). The upper membrane was incubated with anti-His-tag antibodies to detect overexpressed MPP1 and the lower membrane with anti-GAPDH antibodies;
